# Dr. Joel Wilbur Hyde

**Published:** 1907-11

**Authors:** 


### OBITUARY.

Dr. Joel Wilbur Hyde, of Brooklyn, died at his residence in that city September 22, 1907, aged 67 years. He was born at Westbrook, Conn., and received his medical degree from Yale University medical school in 1861. He at once entered the medical service of the army serving as surgeon through the civil war. He then began the practice of his profession in Brooklyn, where he soon achieved prominence, becoming chief medical officer of several life insurance companies. Since 1883, he had been chief of obstetrics in St. Mary's Hospital and was also one of the consulting surgeons in the Bushwick Hospital. He was also consulting obstetrician to the Long Island College Hospital.

